# Enrichment of Bioactive Lipids in Urinary Extracellular Vesicles and Evidence of Apoptosis in Kidneys of Hypertensive Diabetic Cathepsin B Knockout Mice after Streptozotocin Treatment

**DOI:** 10.3390/biomedicines12051038

**Published:** 2024-05-08

**Authors:** Whitney C. Schramm, Niharika Bala, Tanmay Arekar, Zeeshan Malik, Kevin M. Chacko, Russell L. Lewis, Nancy D. Denslow, Yogesh Scindia, Abdel A. Alli

**Affiliations:** 1 Department of Medicine, Division of Nephrology, Hypertension, and Renal Transplantation, College of Medicine, University of Florida, Gainesville, FL 32610, USA; wschramm@stmatthews.edu (W.C.S.); niharikabala@ufl.edu (N.B.); tanmay.arekar@medicine.ufl.edu (T.A.); zmalik456@gmail.com (Z.M.); kchacko@ufl.edu (K.M.C.); yogesh.scindia@medicine.ufl.edu (Y.S.); 2 Department of Physiology and Aging, College of Medicine, University of Florida, Gainesville, FL 32610, USA; 3 Department of Physiological Sciences, Center for Environmental and Human Toxicology, University of Florida, Gainesville, FL 32608, USA; rllewis@ufl.edu (R.L.L.); ndenslow@ufl.edu (N.D.D.); 4 Department of Molecular Genetics and Microbiology, College of Medicine, University of Florida, Gainesville, FL 32608, USA

**Keywords:** cathepsin B, extracellular vesicles, ferroptosis, kidney, lipids, streptozotocin

## Abstract

Cathepsin B (CtsB) is a ubiquitously expressed cysteine protease that plays important roles in health and disease. Urinary extracellular vesicles (uEVs) are released from cells associated with urinary organs. The antibiotic streptozotocin (STZ) is known to induce pancreatic islet beta cell destruction, diabetic nephropathy, and hypertension. We hypothesized that streptozotocin-induced diabetic kidney disease and hypertension result in the release of bioactive lipids from kidney cells that induce oxidative stress and renal cell death. Lipidomics was performed on uEVs isolated from CtsB knockout mice treated with or without STZ, and their kidneys were used to investigate changes in proteins associated with cell death. Lysophosphatidylethanolamine (LPE) (18:1), lysophosphatidylserine (LPS) (22:6), and lysophosphatidylglycerol (LPG) (22:5) were among the bioactive lipids enriched in uEVs from CtsB knockout mice treated with STZ compared to untreated CtsB mice (*n =* 3 uEV preparations per group). Anti-oxidant programming was activated in the kidneys of the CtsB knockout mice treated with STZ, as indicated by increased expression of glutathione peroxidase 4 (GPX4) and the cystine/glutamate antiporter SLC7A11 (XCT) (*n* = 4 mice per group), which was supported by a higher reactivity to 4-hydroxy-2-nonenal (4-HNE), a marker for oxidative stress (*n =* 3 mice per group). Apoptosis but not ferroptosis was the ongoing form of cell death in these kidneys as cleaved caspase-3 levels were significantly elevated in the STZ-treated CtsB knockout mice (*n* = 4 mice per group). There were no appreciable differences in the pro-ferroptosis enzyme acyl-CoA synthetase long-chain family member 4 (ACSL4) or the inflammatory marker CD93 in the kidneys (*n =* 3 mice per group), which further supports apoptosis as the prevalent mechanism of pathology. These data suggest that STZ treatment leads to oxidative stress, inducing apoptotic injury in the kidneys during the development of diabetic kidney disease and hypertension.

## 1. Introduction

Extracellular vesicles (EVs) are nano-sized vesicles secreted by various cell types including renal epithelial cells. EVs have been shown to have beneficial roles and, in other cases, can contribute to pathophysiology [1]. These nano-sized vesicles carry various types of lipids, proteins, and nucleic acids from one cell to another to allow for intercellular communication [2,3] and intracellular signaling [4,5]. The secretion of EVs is regulated by various biological stimuli, drugs, and the post-translational modification or activation/inhibition of proteins. Urinary extracellular vesicles (uEVs) are released from urinary organs including the kidney, urethra, prostate, and bladder [6]. uEVs represent a rich source of biomarkers for various kidney-associated diseases including diabetic kidney disease and hypertension. 

EVs are known to be released by cells undergoing various forms of cell death, including ferroptosis, necroptosis, and apoptosis [7]. Ferroptosis is caused in part by the accumulation of cellular iron and lipid peroxidation under conditions of oxidative stress [8]. Necroptosis is an important factor in many inflammatory diseases associated with inflammation [9]. Apoptosis is a form of programmed cell death mediated by extrinsic or intrinsic pathways that allow for the removal of damaged cells [10]. The biogenesis and release of EVs have been shown to be associated with the export of cellular iron [11]. Wu et al. identified multiple ferroptosis-related genes in syncytiotrophoblast-derived EVs [12]. EVs have been shown to promote necroptosis in various cells. Yelamanchili et al. demonstrated that the necroptotic pathway is activated by EVs enriched in miR-21 and leads to neurotoxicity [13]. Jiao et al. showed that EVs released during hemorrhagic shock promote neutrophil necroptosis [14]. EVs have also been shown to promote apoptosis. The release of cytotoxic EVs can promote apoptosis in various cells. Vennin et al. showed that taxanes cause T cells to release cytotoxic EVs to induce apoptosis in tumor cells but not healthy epithelial cells [15]. Zhou et al. showed that adipocyte-derived EVs induce apoptosis in cumulus cells [16]. Jeon et al. showed that microRNAs enriched in EVs from damaged podocytes induce apoptosis in renal epithelial cells [17]. 

The goals of this study were to investigate whether there are changes in EV lipid profiles and investigate the type of cell death in kidney cells, independent of cathepsin B, after streptozotocin-induced type 1 diabetes and hypertension. 

## 2. Materials and Methods

### 2.1. Chemicals and Reagents

LCMS Optima-grade methylene chloride (DCM), methanol (MeOH), acetonitrile (ACN), and water (H2O) were purchased from Fisher Scientific; Hampton, NH, USA. LCMS-grade isopropanol (IPA) was purchased through HoneyWell (Charlotte, NC, USA). HPLC-grade ammonium acetate was acquired from Fisher Scientific. EquiSplash (Avanti Polar Lipids, Inc.; Birmingham, AL, USA), used as an internal standard, was a mixture of phosphatidylcholine-d7, PC(15:0/18:1-d7); lysophosphatidylcholine-d7, LPC(15:0/18:1-d7); phosphatidylethanolamine-d7, PE(15:0/18:1-d7); lysophosphatidylethanolamine-d7, LPE(15:0/18:1-d7); phosphatidylglycerol-d7, PG(15:0/18:1-d7); phosphatidylinositol-d7, PI(15:0/18:1-d7); phosphatidylserine-d7, PS(15:0/18:1-d7); triacylglyceride-d7, TAG(15:0/18:1-d7/15:0); diacylglyceride-d7, DAG(15:0/18:1-d7); monoacylglyceride-d7, MAG(18:1-d7); cholesterol ester-d7, CE(18:1-d7); sphingomyelin-d9, SM(d18:1/18:1-d9); and ceramide-d7 (d18:1-d7/15:0). The internal standard mix was diluted 100× with 1:1 DCM:MeOH. Bovine heart extract was purchased from Avanti Polar Lipids, Inc.

### 2.2. Animals and Diet

Male and female Cathepsin B knockout mice (B6; 129-Ctsb; Stock No. 030971) were purchased from the Jackson Laboratory (Bar Harbor, ME, USA). Ten-week-old mice were maintained on a Teklad TD 94045 gel diet comprising 0.4% NaCl for a normal-salt diet for the duration of the study. Water was given ad lib. All animal studies were approved by the University of Florida’s Institutional Animal Care and Use Committee. There were *n* = 4 animals in the CtsB knockout mouse group that received the vehicle and *n* = 4 animals in the CtsB knockout mouse group that received STZ. 

### 2.3. Metabolic Cage Studies

Metabolic cage studies were performed using rodent metabolic cages from Ancare (Bellmore, NY, USA), and urine was collected between 3 and 4 pm. Each mouse was housed in a separate metabolic cage.

### 2.4. STZ Injections

Each mouse was restrained and given 200 microliters of STZ (170 mg/kg) intraperitoneally. Treatments were assigned randomly using a pseudo-random number generator for both cohorts. 

### 2.5. Blood Glucose Measurements

Blood glucose was tested by a small tail prick with a 20G needle about 2–3 cm from the tip. A CVS Health Advanced Blood Glucose Meter (Woonsocket, SD, USA) was used to analyze blood glucose once a week before and after the injection of STZ. Mice with a glucose concentration exceeding 300 mg/dL were considered diabetic.

### 2.6. Blood Pressure

The IITC Life Science MRBP System (Woodland Hills, CA, USA) was used to record weekly systolic blood pressure in all mice. A small restrainer recommended for mice up to 40 g was used to carefully restrain the mice without introducing unnecessary stress.

### 2.7. Urinary Extracellular Vesicle Isolation

A total of 10 mL of pooled urine from each mouse was subjected to centrifugation at 1000× *g* for 15 min at 4 °C. Then, a 0.22 µm rapid-flow Nalgene filter (Thermo Fisher Scientific; Waltham, MA, USA) was used to filter each urine sample, and the resulting flow-through was subjected to ultracentrifugation at 52,000 RPM for 90 min at 4 °C utilizing a fixed-angle Ti-70 rotor (Beckman Coulter, Inc; Brea, CA, USA). The EV pellet was washed by first being reconstituted in 500 μL of ultrapure 1XPBS and then further diluted in 30 mL of ultrapure 1XPBS before it was subjected to another round of ultracentrifugation at the same speed and for the same duration. Finally, the EV pellet was resuspended in 200 μL of ultrapure 1XPBS (ThermoFisher Scientific) and stored at −80 °C. There were *n* = 3 uEV preparations for each of the two animal groups. 

### 2.8. Nanoparticle Tracking Analysis

A nanoparticle tracking analysis was performed using an NS-300 NanoSight machine (Salisbury, UK). A small EV sample suspended in ultrapure 1XPBS and diluted 1:1000 was subjected to a nanoparticle tracking analysis in order to determine the uEV concentration and size. 

### 2.9. BCA Assay and Western Blotting

Nine BSA standards, prepared via serial dilutions from a 2 mg/mL stock solution of BSA (Sigma-Aldrich, St. Louis, MO, USA) and a BCA reagent assay kit (ThermoFisher Scientific, Waltham, MA, USA), were used to determine the total protein concentration of tissue lysate sample from CtsB knockout mice by first plotting a linear regression curve from the optical density readings at 570 nm for the standards and then calculating the concentrations of each sample. Fifty micrograms of total protein were then loaded onto 4–20% Tris Glycine gels (ThermoFisher Scientific) and resolved at 200 volts for 1 h on a BioRad Criterion system (Hercules, CA, USA). A BioRad Criterion system was also used to electrically transfer the proteins onto nitrocellulose membranes (ThermoFisher Scientific) for 4 h in chilled Towbin buffer. A solution of 5% (*w*/*v*) nonfat dry milk prepared in 1XTBS was used to block the membranes for 1 h at room temperature before they were incubated with primary antibodies (Table 1). After a series of 1XTBS washes, the blots were incubated with a goat anti-rabbit secondary antibody at room temperature. The blots were incubated with ECL Select Western blotting detection reagent (GE Healthcare; Amersham, UK) for 5 min and then imaged on a ChemiDoc XRS imaging system (BioRad; Hercules, CA, USA).

### 2.10. Lipid Extraction

Lipids were extracted from the EVs using the Bligh and Dyer method as described by Bligh, E.G. and Dyer, W.J. [18]. A volume of 12 to 40 µLEVs of each sample, measured to equal 40 µg of protein, was used. EVs were first normalized to 40 µg of protein and then brought up to a 1 mL volume of water and allowed to equilibrate for 10 min on ice. Chilled MeOH (2 mL) and DCM (0.9 mL) were added and vortexed; 5 µL of 1 µg/mL EquiSplash was then added using a Hamilton syringe and vortexed. The samples were then incubated at room temp for 30 min before 1 mL of H2O and 0.9 mL of DCM were added and vortexed. The samples were then centrifuged at 1200 RPM for 10 min at room temp to separate the organic and aqueous phases. The bottom organic phase was removed and placed into separate clean glass tubes using a long-tip Pasteur pipette. The samples were re-extracted by adding 2 mL of DCM, vortexed, and centrifuged. The bottom organic layers from the two extractions were pooled with the previous organic layer. The samples were evaporated under nitrogen gas at room temp. The samples were reconstituted in 100 µL of Solvent A (7:93 DCM:ACN; 2 mM ammonium acetate).

### 2.11. LC-MS/MS

An ultra-high-performance liquid chromatography Nexera X2 (UHPLC, Shimadzu Co.; Tampa, FL, USA) system coupled to a QTrap 6500 mass spectrometer (AB Sciex, Redwood Shores; Farmingham, MA, USA) was used to analyze the lipid samples. Chromatographic separation was performed under normal phase conditions utilizing a Luna 3 µm NH2, 2 × 100 mm (Phenomenex; Torrance, CA, USA) column. A binary gradient was used over 17 min, using 7:93 DCM:ACN and 2 mM ammonium acetate as mobile phase A and 50:50 H2O:ACN and 2 mM ammonium acetate as mobile phase B. Finally, 100% IPA was used as a needle wash solution.

### 2.12. Lipid Quantitation

A total of 20 lipid groups, including SM, CE, CER, glucosylceramide (GlcCER), lactosylceraminde (LacCER), PE(P-), TAG, DAG, MAG, LPC, PC, LPE, PE, PE(O-), LPG, PG, LPI, PI, LPS, and PS, were acquired using Analyst 1.7 Hotfix 3 software. The quantitation of lipid concentrations was conducted by quantitating each lipid class with the associated lipid IS class area and concentration. MultiQuant (ver 3.0.3) software was used for quantitation. Instrument performance was validated by analyzing bovine heart extract. Since the samples were pre-normalized by aliquoting 40 µg of protein, post-processing normalization was not required.

### 2.13. Immunohistochemistry

Formalin-fixed kidney tissues from CtsB knockout mice treated with or without STZ cut to a thickness of 4 µm were subjected to 2 exchanges of xylene (Fisher Scientific) and then a series of ethanol exchanges for 3 min each (twice in 100% ethanol, once in 95% ethanol, and once in 50% ethanol). Next, the tissues were washed with type 1 water for 3 min before they were placed in boiling citrate buffer for 20 min. After being washed with type 1 water for 3 min, the tissues were placed in 1XPBS (Corning, Manassas, VA, USA) for 5 min before they were incubated in a blocking solution (2.5% normal horse serum (Vector Laboratories, Inc.; Newark, CA, USA) for 20 min in a humidified chamber. Afterwards, the tissues were incubated with a 1:250 dilution of primary antibody (rabbit polyclonal) (Table 1) prepared in a blocking solution for 60 min in a humidified chamber. After a wash with 1XPBS, the tissues were incubated with the second primary antibody (mouse monoclonal) (Table 1) for 60 min in a humidified chamber. After 3 exchanges with 1XPBS, each lasting 2 min, the tissues were incubated with VectaFluor Duet reagent (Vector Laboratories, Inc.) for 30 min in a humified chamber. Next, the tissues were subjected to 3 exchanges of 1XPBS, each lasting 2 min. Finally, one drop of Vectashield anti-fade mounting media with DAPI (Vector Laboratories, Inc.) was added to the tissues before the application of a 22X22-1 glass coverslip (Fisher Scientific). 

### 2.14. Data Analysis and Statistics

Student’s *t*-test in SigmaPlot software version 14.5 (Jandel Scientific, San Rafael, CA, USA) was used to determine statistical significance between the two groups. A Mann–Whitney U test was performed if the data from the two independent groups were not normally distributed. Results were considered significant at a *p*-value < 0.05.

## 3. Results

### 3.1. STZ Increases Blood Glucose and Blood Pressure in CtsB Knockout Mice

Cathepsin B is known to cleave and activate epithelial sodium channels (ENaCs) in the kidney [19]. Renal ENaC activity augmented by cathepsin B was shown to lead to an increase in sodium retention and hypertension in mice with nephrotic syndrome [19]. Here, we investigated for the first time whether blood glucose and blood pressure are augmented in adult CtsB knockout mice after STZ treatment. Compared to untreated CtsB knockout mice, CtsB mice treated with a single dose of STZ showed a significant increase in both blood glucose and blood pressure (Table 2).

### 3.2. Characterization of uEVs from CtsB Knockout Mice Treated with or without STZ

**Figure 1:**
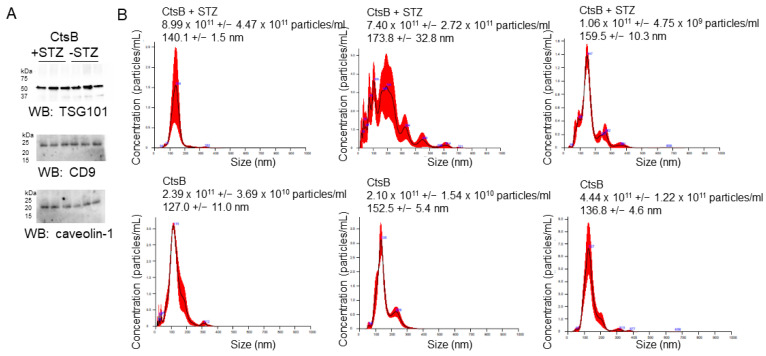
Characterization of urinary extracellular vesicles (uEV) from CtsB knockout mice treated with or without streptozotocin (STZ). (**A**) Western blot analysis of established EV markers (TSG101, CD9, and caveolin-1) from uEVs isolated from CtsB knockout mice treated with or without STZ. (**B**) Nanoparticle tracking analysis showing uEV concentration and size; *n* = 3 uEV preparations per group.

The enrichment of multiple EV markers in uEVs from CtsB knockout mice treated with or without STZ was assessed by Western blotting. TSG101, CD9, and caveolin-1 were all found to be enriched in each uEV preparation from the two groups (Figure 1A). A nanoparticle tracking analysis showed that each EV preparation contained predominately smaller EVs with a diameter of less than 150 nm (Figure 1B).

### 3.3. Lipidomic Analysis of uEVs from CtsB Knockout Mice Treated with or without STZ

**Figure 2:**
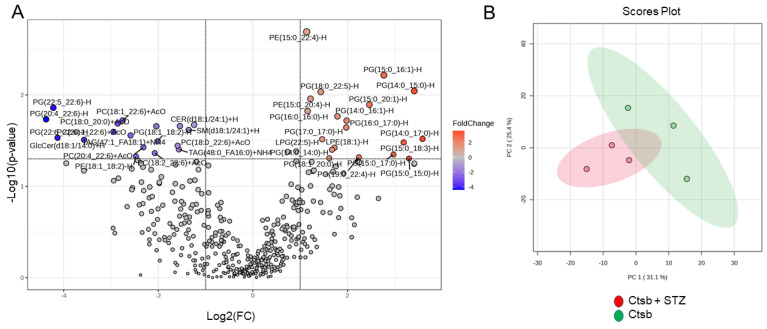

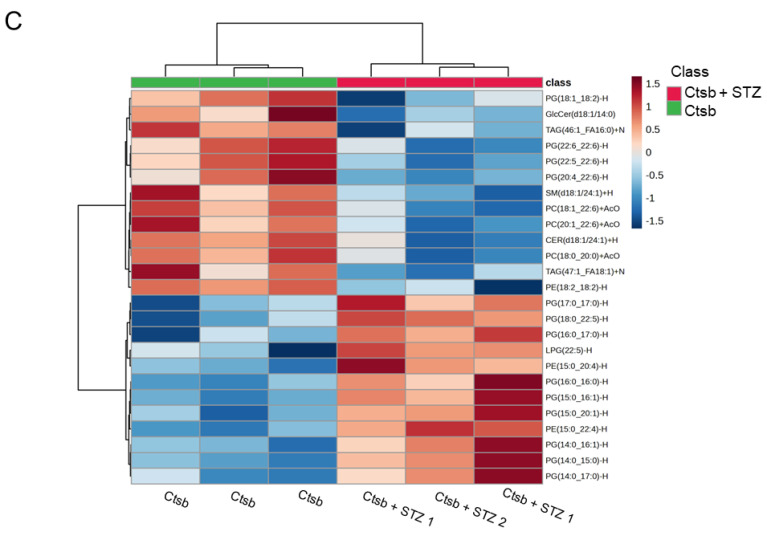
A lipidomic analysis of uEVs from CtsB knockout mice treated with or without STZ. (**A**) A volcano plot showing 18 significantly downregulated lipids and 19 significantly upregulated lipids in the kidneys of STZ-treated CtsB knockout mice compared to untreated CtsB knockout mice. Grey dots represent the lipids that were comparable between the two groups. (**B**) Principal component analysis 2D score plot displaying 95% confidence regions. (**C**) A heatmap showing Euclidean distance measurements; the Ward clustering method showing the top 25 lipids that showed a significant difference between the two groups, with each colored cell on the map corresponding to a concentration value. *n* = 3 per group. After removing missing values, 426 lipid species were analyzed. An interquartile range variance filter and a mean intensity value abundance filter were used for data filtering. The dataset was normalized using the median with a log transformation and autoscaling.

uEVs are known to be enriched in various signaling and bioactive lipids that play a role in pathophysiology. Here, we investigated the enrichment of different lipid classes and lipid species in uEVs from CtsB knockout mice treated with or without STZ. From a total of 426 lipids that were detected in each uEV preparation sample in the two groups, 18 lipids were significantly downregulated while 19 lipids were significantly upregulated in the CtsB knockout mice that received STZ compared to the untreated CtsB knockout mice (Figure 2). 

### 3.4. Detection of Bioactive Lipids in uEVs from CtsB Knockout Mice Treated with or without STZ

**Figure 3:**
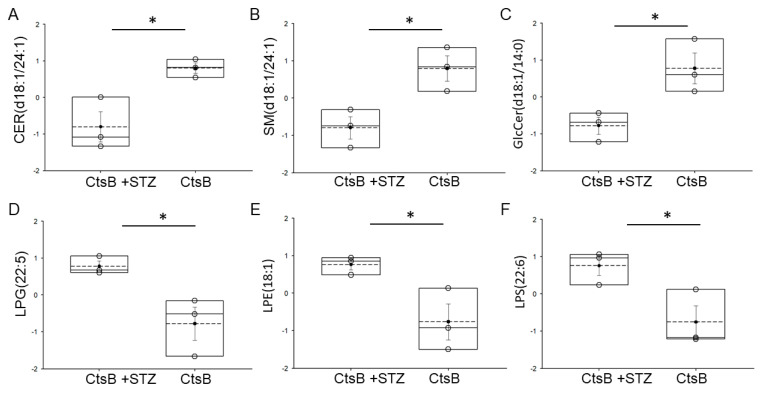
Bioactive lipids detected in uEVs from cathepsin B knockout (CtsB) mice treated with streptozotocin (STZ) compared to untreated CtsB knockout mice. Box plot showing lipids that were significantly different between the two groups. (**A**) CER(d18:1/24:1); (**B**) SM (d18:1/24:1); (**C**) GlcCer(d18:1/14:0); (**D**) LPG(22:5); (**E**) LPE(18:1); (**F**) LPS(22:6). Lipids were normalized to median. * represents a *p*-value of < 0.05. The boxplot shows the mean (dotted line), median (solid line), and interquartile ranges.

Six different bioactive lipids were detected in uEVs from CtsB knockout mice treated with or without STZ (Figure 3). CER(d18:1/24:1), SM(d18:1/24:1), and GlcCer(d18:1/14:0) were the three bioactive lipids that were downregulated in the CtsB knockout mice treated with STZ compared to the untreated CtsB knockout mice (Figure 3). There were increases in three bioactive lipids, lysophosphatidylethanolamine (LPE) (18:1), lysophosphatidylserine (LPS) (22:6), and lysophosphatidylglycerol (LPG) (22:5), in the uEVs from the STZ-treated CtsB knockout mice compared to the uEVs from the untreated CtsB knockout mice (Figure 3). 

### 3.5. STZ Induces 4-HNE Upregulation in the Kidneys of CtsB Knockout Mice

**Figure 4:**
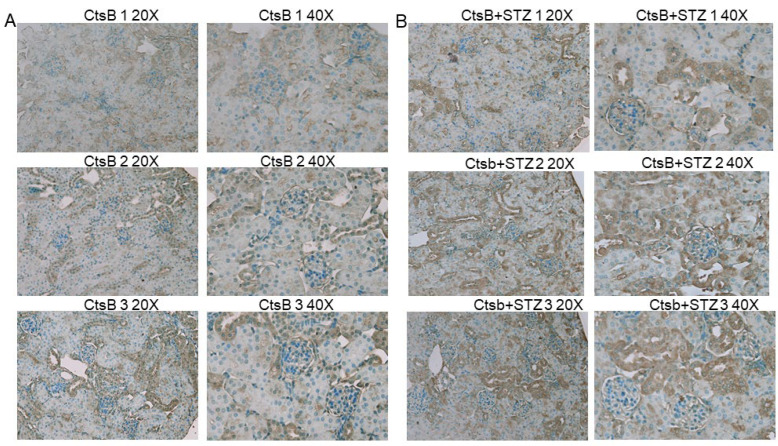
4-hydroxy-2-nonenal (4-HNE) staining in the kidneys of Ctsb knockout mice treated with or without STZ. (**A**) 4-HNE staining in CtsB mice treated without STZ and (**B**) 4-HNE staining in CtsB mice treated with STZ. Images taken at both 20× and 40× magnification are shown. *n* = 3 per group.

4-hydroxy-2-nonenal (4-HNE) is a known mediator of oxidative stress and cell injury in cells and tissues. Thus, we investigated whether kidneys from CtsB knockout mice treated with STZ had increased 4-HNE staining. The STZ-treated CtsB knockout mice showed more positive 4-HNE staining in renal tubules compared to untreated CtsB knockout mice (Figure 4). 

### 3.6. STZ Treatment of CtsB Knockout Mice Results in Increases in Renal GPX4 and XCT Protein Expression 

**Figure 5:**
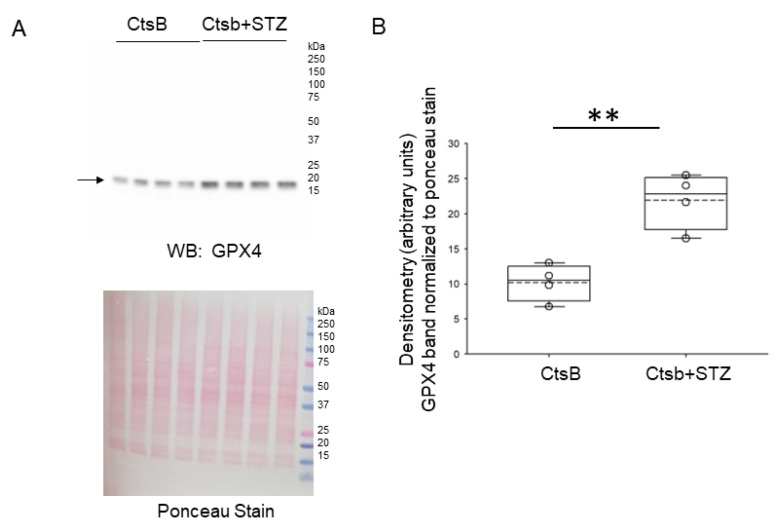
Western blot analysis of GPX4 protein expression in kidney lysates from cathepsin B knockout (CtsB) mice treated with or without streptozotocin (STZ). (**A**) Western blot of GPX4 (top) Ponceau staining (bottom) was used to assess lane loading; (**B**) densitometric analysis of the GPX4 band normalized to Ponceau. ** represents *p*-value of < 0.01. The boxplot shows the mean (dotted line), median (solid line), and interquartile ranges.

**Figure 6:**
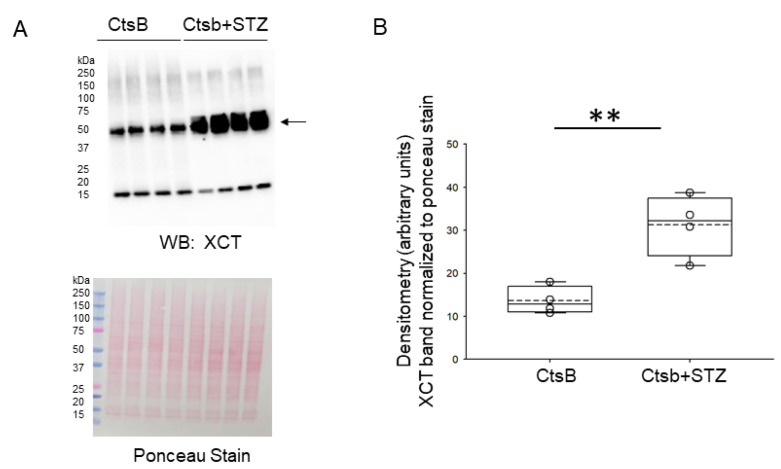
Western blot analysis of XCT protein expression in kidney lysates from cathepsin B knockout (CtsB) mice treated with or without streptozotocin (STZ). (**A**) Western blot of XCT (top); Ponceau staining (bottom) was used to assess lane loading. (**B**) Densitometric analysis of the XCT band normalized to Ponceau. ** represents *p*-value of < 0.01. The boxplot shows the mean (dotted line), median (solid line), and interquartile ranges.

Next, we investigated the expression of proteins indicative of an ongoing anti-oxidative stress program in the kidneys of CtsB knockout mice treated with or without STZ by Western blotting. Compared to untreated CtsB mice, there was a significant increase in the renal expression of GPX4 (Figure 5) and the cystine/glutamate antiporter SLC7A11 (XCT) of the STZ-treated CtsB knockout mice (Figure 6). 

### 3.7. STZ Treatment Activates Caspase but Not ACSL4 in the Kidneys of CtsB Knockout Mice 

**Figure 7:**
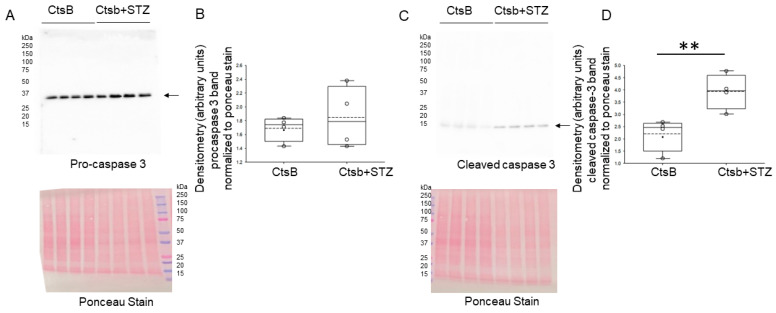
Western blot analysis of pro-caspase 3 and cleaved caspase-3 in kidney lysates from cathepsin B knockout (CtsB) mice treated with or without streptozotocin (STZ). (**A**) Western blot of pro-caspase 3 (top). Ponceau staining (bottom) was used to assess lane loading; (**B**) densitometric analysis of the pro-caspase 3 band normalized to Ponceau. (**C**) Western blot of cleaved caspase-3 (top). Ponceau staining (bottom) was used to assess lane loading; (**D**) densitometric analysis of the cleaved caspase 3 band normalized to Ponceau. ** represents *p*-value of < 0.01. The boxplot shows the mean (dotted line), median (solid line), and interquartile ranges.

The activation of the lipid peroxidation enzyme acyl-CoA synthetase long-chain family member 4 (ACSL4) results in the amplification of lipid peroxidation and the induction of ferroptosis. We investigated whether STZ treatment augments the ACSL4 protein in the kidney. An immunohistochemistry fluorescence microscopy analysis showed no appreciable difference in ACSL4 protein expression in kidney sections from CtsB knockout mice treated with or without STZ (Supplementary Figure S1). The lack of inflammation, as evidenced by the CD93 staining (Supplementary Figure S2) of kidney sections from CtsB knockout mice treated with or without STZ (Supplementary Figure S2), further supported a non-ferroptotic mechanism of cell death. Due to the lack of inflammation, we probed the kidneys for pro-caspase 3 and cleaved caspase-3 as non-inflammatory forms of cell death. The protein expression levels of pro-caspase 3 in the kidneys of the CtsB knockout mice treated or without STZ (Figure 7) were comparable. However, the protein expression level of cleaved caspase 3 was augmented in the kidneys of the CtsB knockout mice treated with STZ compared to the untreated CtsB knockout mice (Figure 7).

## 4. Discussion

Phospholipids such as phosphatidylglycerols (PGs) have been shown to play an important role in disease mechanisms. Palmitoyl-oleoyl molecular species of PG (POPGs) have been shown to be a regulator of innate immunity and viral infection [20]. Kandasamy et al. showed that PGs derived from pulmonary surfactant suppress *M. pneumoniae* induced arachidonic acid release from mouse macrophage cells [21]. PG production was shown by Kayser et al. to be induced by gut dysbiosis and positively correlated with the remodeling of adipose tissue during obesity [22]. A study by Zeng et al. showed that PGs, among other lipids, were downregulated in the renal cortex and medulla regions of the kidneys of mice with acute cadmium-induced nephrotoxicity [23]. Kurano et al. showed increased levels of several PGs detected in urine samples from patients with severe COVID-19 during its later phase [24]. A previous study by our group showed that PG levels were greater in EVs isolated from the basolateral compartment compared to the apical compartment of cultured mouse cortical collecting duct cells [25]. In this study, PGs comprised one of the largest classes of phospholipids that showed differences in the amount enriched in uEVs from CtsB knockout mice after STZ-induced diabetes and hypertension. In addition, longer PGs were found to be downregulated in uEVs from CtsB knockout mice treated with STZ compared to healthy CtsB knockout mice. These data suggest that there is a reduction in carbon length in PGs after STZ treatment.

A lipidomic analysis of uEVs from salt-loaded hypertensive wild-type 129Sv mice has already been reported by Chacko et al. [26]. In that study, the effect of the antioxidant tempol on uEV lipids was assessed. There were nine PGs that were upregulated and thirteen PGs that were downregulated after tempol treatment. There were three LPEs that were upregulated and two LPEs that were downregulated. While there are similar trends in which specific lipid classes are enriched in uEVs from hypertensive 129Sv mice and STZ-induced diabetic and hypertensive CtsB knockout mice, the differences in lipid species can be attributed to genotypic differences between the mice since CtsB knockout mice are from a mixed B6/129 background. 

Our group and others have shown that ferroptosis-associated proteins are enriched in uEVs. For example, in some studies, caveolin-1 has been shown to have a protective role against ferroptosis. Deng et al. showed that caveolin-1 protects hepatocytes against ferroptosis in hepatitis mediated by autoimmunity [27]. Yang et al. showed that caveolin-1 suppresses autophagy-dependent ferroptosis and alleviates the formation of calcium oxalate kidney stones [28]. In another study, Caveolin-1 was shown to inhibit ferroptosis and promote cancer progression [29]. Our group previously showed that caveolin-1 is enriched in uEVs isolated from the urine of aged, spontaneously hypertensive mice [30]. Interestingly, a study by Lugo et al. showed a greater enrichment of caveolin-1 in uEVs from diabetic db/db mice administered hAAT compared to a vehicle [31]. Other groups have investigated the correlation between caveolin-1 and phosphatidylglycerol levels. Qin and Bollag investigated whether a caveolin-1 scaffolding domain reduce the levels of phophatidylglycerol and inhibits calcium-induced differentiation in mouse keratinocytes [32]. The data from that study suggested that the caveolin-1 scaffolding domain alters lipid rafts in keratinocytes. In the present study, we did not observe any appreciable changes in caveolin-1 enrichment in uEVs from CtsB knockout mice treated with or without STZ. These data suggest that the pathology associated with STZ is not attributed to ferroptosis. 

To further investigate whether STZ administration induces ferroptosis, we measured the protein expression of multiple ferroptosis-associated proteins in the native kidney or kidney lysates from CtsB knockout mice treated with or without STZ. First, there was an increase in 4-hydroxy-2-nonenal (4-HNE) in the kidneys of CtsB knockout mice treated with STZ, which may be associated with increased oxidative stress. The expression levels of both GPX4 and CTX were augmented in the kidney lysates of STZ-treated CtsB knockout mice compared to untreated CtsB knockout mice. Both GPX4 and CTX were previously shown to be downregulated in models of ferroptosis [33,34]. Since ACSL4 is known to be a pro-ferroptotic gene [35,36] and its activation is known to be essential in the execution of ferroptosis, we investigated whether STZ treatment results in an increase in ACSL4 protein expression in the kidney. However, there were no appreciable differences in ACSL4 protein expression in the kidneys of the two groups. These data further suggested that STZ treatment does not induce ferroptosis in the CtsB knockout kidney.

To investigate whether STZ induces another form of renal cell injury, we investigated whether there is an activation of caspase 3 in the kidneys of CtsB knockout mice treated with STZ. Western blot and densitometric analyses showed increased levels of cleaved caspase-3 in the kidneys of STZ-treated CtsB knockout mice, indicating the activation of the caspase 3 pathway. These data suggest that STZ may induce renal cell injury and apoptosis, which may contribute to the development of diabetic kidney disease and hypertension in these animals. 

**Figure 8:**
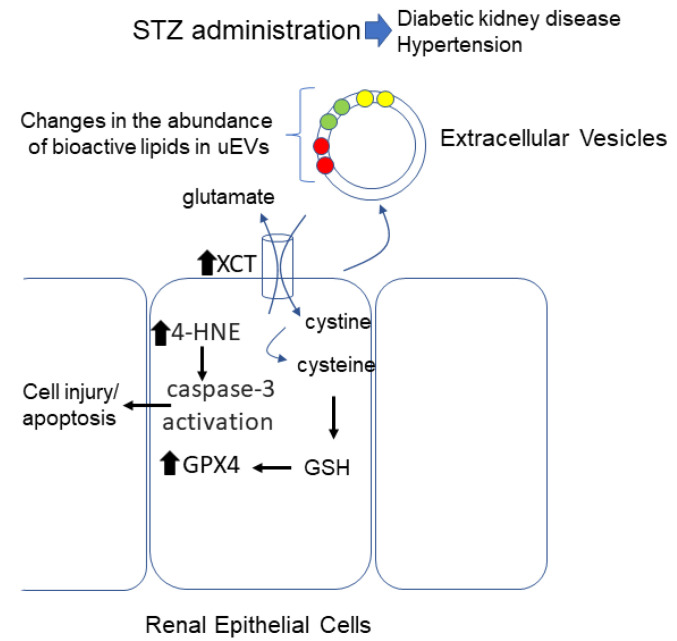
Summary of key results after treating CtsB knockout mice with STZ. CtsB knockout mice treated with STZ compared to untreated CtsB mice release EVs enriched in bioactive lipids lysophosphatidylethanolamine (LPE) (18:1), lysophosphatidylserine (LPS) (22:6), and lysophosphatidylglycerol (LPG) (22:5). Kidneys from STZ-treated CtsB knockout mice have elevated levels of 4-HNE and caspase-3 activation. In addition, kidneys of CtsB knockout mice treated with STZ show increased expression of XCT and GPX4 proteins. Red filled circles represent LPG (22:5), green filled circles represent LPE(18:1), and yellow filled circles represent LPS(22:6). Black filled arrows represent increased protein expression.

Although our study introduces several novel findings associated with the effects of STZ on the kidney (Figure 8), there are some limitations. One limitation of this study is that we did not compare PE, PS, and PG levels in the kidneys of animals receiving acute and chronic STZ treatment. A second limitation is that we did not examine regional differences in lipids in the kidneys of mice that were administered STZ or the vehicle. It is possible that the levels of bioactive lipids including LPEs, LPSs, and LPGs change over time and show differences between the cortex and medulla. In order to be able to determine whether specific bioactive lipid species can serve as kidney injury markers, these studies are necessary. Finally, we did not investigate whether uEVs released from kidney epithelial cells after treating CtsB knockout mice with STZ induce apoptosis in different cell types within specific segments of the nephron.

Future experiments will investigate whether there are differences in the organization of the actin cytoskeleton after treating proximal tubule and distal tubule epithelial cells with uEVs from STZ-treated CtSB knockout mice compared to uEVs from wild-type mice. These studies will demonstrate the functional role of cathepsin B in regulating actin cytoskeleton dynamics. 

## 5. Conclusions

Here, for the first time, we investigated changes in the lipid profiles of uEVs and cell death in the kidneys of CtsB knockout mice treated with or without STZ. A lipidomic analysis of the uEVs from STZ-treated CtsB knockout mice revealed that PEs and PGs are the two classes of lipids that are the most sensitive to changes after STZ treatment and that there was an enrichment of bioactive lipids, including the lipids LPE18:1, LPS22:6, and LPG22:5, in uEVs from the STZ group. Taken together, our data suggest that STZ does not cause iron-dependent necrosis induced by lipid peroxidation in the kidneys of CtsB knockout mice but instead may cause apoptosis associated with an increase in oxidative stress.

## Figures and Tables

**Table 1:** Antibodies used in this study.

Antibody	Company	Catalog Number	Application
caveolin-1	Cell Signaling Tech; Danvers, MA, USA	3267	Western blotting
CD9	Abcam; Waltham, MA, USA	ab223052	Western blotting
TSG101	Abcam; Waltham, MA, USA	ab30871	Western blotting
GPX4	Abcam; Waltham, MA, USA	ab125066	Western blotting
XCT	Abcam; Waltham, MA, USA	ab175186	Western blotting
ACLS4	Santa Cruz Biotech; Dallas, TX, USA	Sc-271800	Immunohistochemistry
CD93	Santa Cruz Biotech; Dallas, TX, USA	Sc-365172	Immunohistochemistry
Caspase-3	Proteintech; Rosemont, IL, USA	66470	Western blotting
Cleaved caspase-3	Cell Signaling Tech; Danvers, MA, USA	9661	Western blotting
4-Hydroxynonenal	R&D Systems; Minneapolis, MN, USA	MAB3249	Immunohistochemistry

**Table 2:** CtsB mice treated with or without STZ. Both cohorts of mice had comparable body weights (*n* = 4 mice per group).

Parameter	CtsB STZ	CtsB	*p*-Value
Body weight	25.065	27.148	*p* = 0.549
Blood glucose	396.250	149.750	*p* = 0.001
Blood pressure	139.250	105.000	*p* ≤ 0.001

## Data Availability

The lipidomic dataset presented in this study is openly available via the following FigShare link: https://figshare.com/s/1ec55562544dcc5ca574 (accessed on 8 December 2023). Other data presented in this study are available upon request from the corresponding author.

## Supplementary Materials

The following supporting information can be downloaded at: https://www.mdpi.com/article/10.3390/biomedicines12051038/s1. Figure S1. Immunohistochemistry analysis of ACSL4 in the kidneys of CtsB knockout mice treated with or without STZ. The top row shows positive signal (bright green) for CtsB knockout mice treated with STZ. The bottom row shows positive signal for untreated CtsB knockout mice. Images were taken using a 40× objective. N = 3 per group. Figure S2. Immunohistochemistry analysis of CD93 in the kidneys of CtsB knockout mice treated with or without STZ. The top row shows positive signal (bright red) for CtsB knockout mice treated with STZ. The bottom row shows positive signal for untreated CtsB knockout mice. Images were taken using a 40× objective. N = 3 per group.
