# Supplementary material for: Enrichment of Bioactive Lipids in Urinary Extracellular Vesicles and Evidence of Apoptosis in Kidneys of Hypertensive Diabetic Cathepsin B Knockout Mice after Streptozotocin Treatment
Source: Biomedicines. 2024 May 8;12(5):1038. doi: 10.3390/biomedicines12051038 (PMC11117475; doi:10.3390/biomedicines12051038)
Supplement: Supplementary file 1 [file biomedicines-12-01038-s001.zip › biomedicines-2877570-supplementary.pdf]

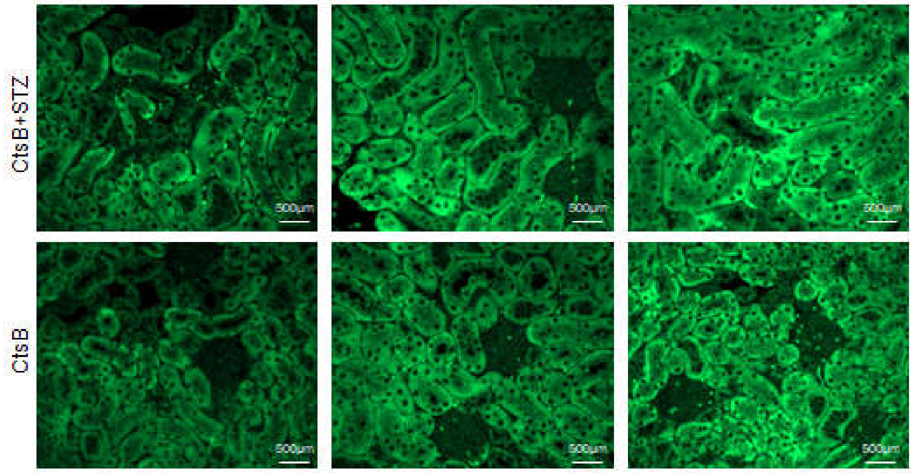

Supplementary Figure S1. Immunohistochemistry analysis of ACSL4 in the kidneys of CtsB knockout mice treated with or without STZ. The top row shows positive signal (bright green) for CtsB knockout mice treated with STZ. The bottom row shows positive signal for untreated CtsB knockout mice. Images were taken using a 40X objective. N=3 per group.

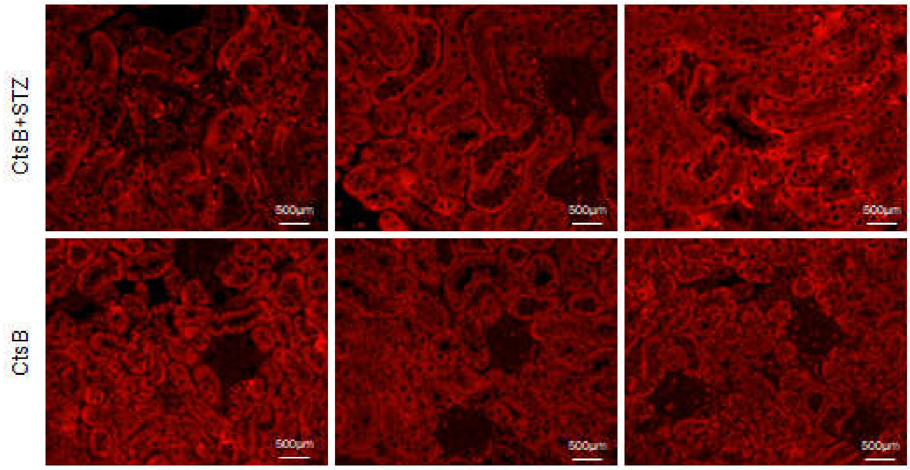

Supplementary Figure S2. Immunohistochemistry analysis of CD93 in the kidneys of CtsB knockout mice treated with or without STZ. The top row shows positive signal (bright red) for CtsB knockout mice treated with STZ. The bottom row shows positive signal for untreated CtsB knockout mice. Images were taken using a 40X objective. N=3 per group.
